# Super-resolution Fluorescence Imaging of Recycled Polymer Blends via Hydrogen Bond-Assisted Adsorption of a Nile Red Derivative

**DOI:** 10.1021/acs.langmuir.3c01976

**Published:** 2023-10-03

**Authors:** Chao-Chun Hsu, Markus Rückel, Daniel Bonn, Albert M. Brouwer

**Affiliations:** † van’t Hoff Institute for Molecular Sciences, University of Amsterdam, Science Park 904, 1098 XH Amsterdam, The Netherlands; ‡ Group Research, BASF SE, Ludwigshafen D-67056, Germany; § van der Waals-Zeeman Institute, Institute of Physics, University of Amsterdam, Science Park 904, 1098 XH Amsterdam, The Netherlands

## Abstract

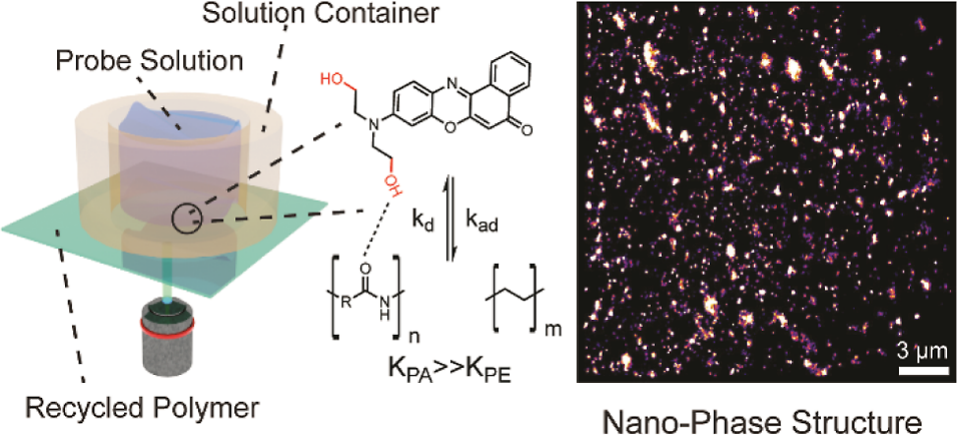

A key challenge in the recycling of multilayer plastic films of polyethylene and polyamide, as typically used for food packaging, is to assess and control the phase separation of the two types of polymers in the recycled material, the specifics of which determine the mechanical strength of the recycled material. However, visualizing the polyamide-in-polyethylene domains with conventional fluorescence methods or electron microscopy is challenging. We present a new approach that combines the point accumulation in nanoscale topography (PAINT) super-resolution method with a newly synthesized Nile Red probe (diOHNR) as the fluorescent label. The molecule was modified to undergo a hydrogen bond-assisted interaction with the polyamide phase in the blend due to its two additional hydroxyl groups but preserves the spectral properties of Nile Red. As a result, the localization density of the probe in the PAINT image is 13 times larger at the polyamide phase than at the polyethylene phase, enabling quantitative evaluation of the spatial polyamide/polyethylene distribution down to the nanoscale. The method achieved a spatial resolution of 18.8 nm, and we found that over half of the polyamide particles in a recycled sample were smaller than the optical diffraction limit. Being able to image the blends with nanoscopic resolution can help to optimize the composition and mechanical properties of recycled materials and thus contribute to an increased reuse of plastics.

## Introduction

The concept of polymers was first introduced 100 years ago.^1^ Over the past 50 years, single-use polymer-based materials have become an integral part of our daily lives due to their convenience and low cost.^2,3^ Since then, 7.4 billion metric tons of plastic have been left in the Earth’s system, plus about 10 million tons of plastic directly disposed of in the ocean.^4^ These plastics are highly persistent and will take hundreds of years to degrade in ambient conditions, while further threatening the biosphere by producing toxic components during decomposition.^5^ The particles in the ocean can accumulate in organisms, potentially affecting the ecological system.^6^ To address this issue, a waste management strategy summarized as “reduce, reuse, recycle, and recover” (4Rs) was proposed.^7,8^ Here we focus on the recycling of plastics typically used for food packaging, consisting of multilayers of polyethylene (PE) and polyamide (PA). Such plastics were classified as “non-recyclable” due to the assumed incompatibility between PA and PE. However, a systematic study revealed that, if PA content is <10%, PA is evenly dispersed in the PE matrix, and for higher PA contents, compatibilizers can be added to enable the dispersion of PA into the PE phase during the mechanical extrusion process.^9,10^ Not only do these compatibilizers render the PE waste stream mechanically recyclable but they also bolster the mechanical properties of the film. By blending “hard” PA into PE, which is prone to weakening due to chain scission during the mechanical extruding, the durability of the film is enhanced.^7,11,12^

The mechanical strength of recycled materials strongly depends on the average domain size of the PA inside the continuous PE phase: the smaller the PA particles, the more robust and recyclable the materials become.^9,13−16^ Hence, characterizing and visualizing the size distribution of the (nanoscale) PA domains in PE can help to design better compatibilizers for blending. Scanning electron microscopy (SEM) can be used to reveal the topographical morphology of the PA/PE blend by measuring scattered electrons from the surface. However, it does not provide direct information about PA domains.^13,17^ Transmission electron microscopy (TEM) is another method that can be used to visualize the PA phase by staining the PA with phosphotungstic acid, which gives high contrast in TEM images due to the heavy atom.^16,18^ However, TEM suffers from challenging sample preparation as the polymer needs to be sliced into a thickness of less than 100 nm. This is sometimes smaller than the domain size of PA, and thus, the PA may be removed during the sample preparation.

**Figure 1:**
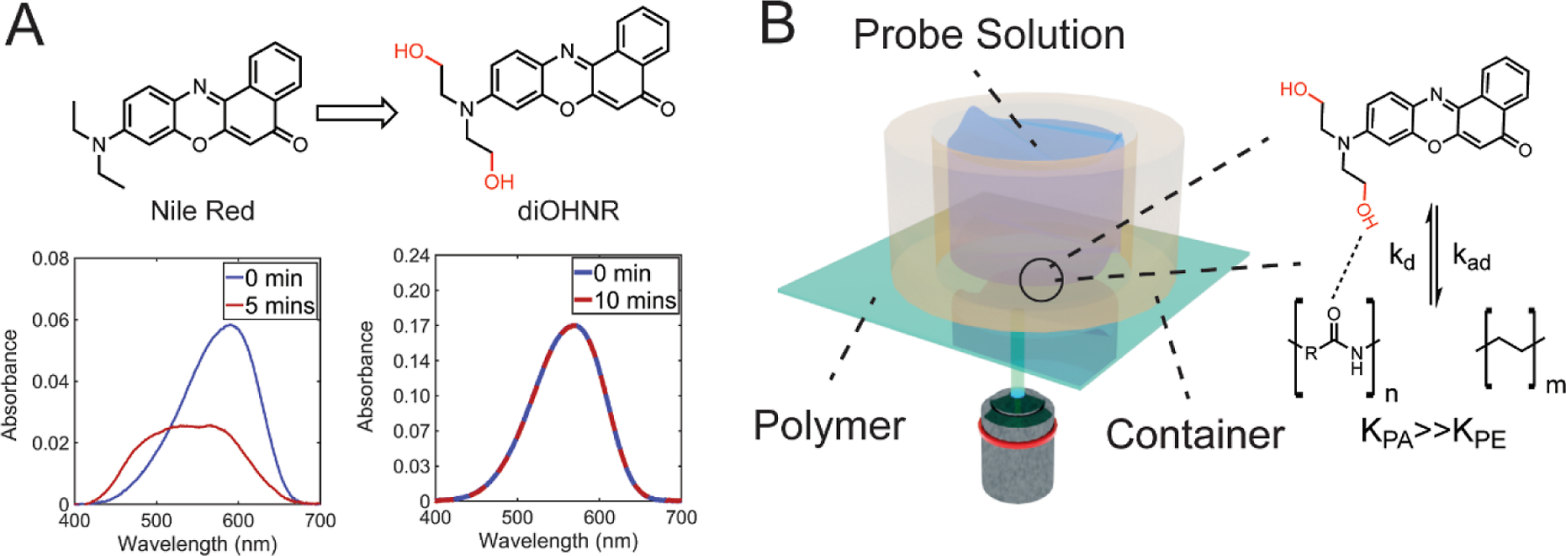
(A) Chemical structures of commercially available Nile Red (top left) and diOHNR (top right). Unmodified Nile Red (2 μM) aggregates within 5 min in water, as shown by the drastic spectral change (bottom left), while the addition of two hydroxyl groups improves its solubility in aqueous solutions (bottom right). (B) Illustration of the principle of “chemical” phase mapping. Single-molecule fluorescence images are recorded in total internal reflection fluorescence mode. The detailed setup can be found in the Supporting Information. The probe, dissolved in the solution (<1 nM), has a higher affinity toward the PA phase, resulting in a higher localization density. The equilibrium constants (*K*) determine the probe’s efficiency in distinguishing phases in the super-resolution image.

In this study, to overcome the drawbacks of electron microscopy, we utilized fluorescence microscopy. Recent developments in optical setups and fluorescent probes have enabled fluorescence microscopy to reach subnanoscopic scales while offering three-dimensional information.^19−23^ The MINFLUX/SIMFLUX method can even achieve a resolution of 1 nm, comparable to electron microscopy.^24−26^ In the field of polymer science, the development of single-molecule localization microscopy (SMLM) has been slower compared to the explosive growth in biological research.^27^ Ross et al. were the first to selectively functionalize one of the polymers with a fluorophore in a mixture and use SMLM to distinguish the phase structure.^28^ Wöll et al. used photoswitchable dyes to visualize the block copolymer phase structure.^29^ Ito et al. further used the technique to study the diffusion coefficients of polymers.^30^ However, these methods require the chemical anchoring of the probe during the polymerization process, which cannot be fulfilled for recycled materials. To address this, we rely on the surface-sensitive point accumulation for imaging in nanoscale topography (PAINT) method introduced by Hochstrasser and Sharonov,^31^ as illustrated in Figure 1B. Dyes in a diluted solution (usually in the nanomolar range) adsorb at different phases. The fluorescence of a single molecule can be localized because the diffusion coefficient of the dye at the surface is much smaller than that in the solution.^32^ Moreover, the probe used in the measurement has a low quantum yield in the aqueous solution but emits more efficiently when bound to a surface.^33,34^ PAINT offers the specific advantage of not requiring labeling or anchoring points, so the method does not disturb the target.^35^ PAINT is widely been used in bioimaging; for example, in the DNA–PAINT method, a fluorescent oligonucleotide binds to its complementary target strands to reveal the structures of the targets.^36,37^ In the study of polymer phase structures, Zhang et al. used randomly adsorbed probe molecules to distinguish the surface structure of the metal–polymer; the metal surface quenches fluorescence while the polymer enhances single-molecule fluorescence.^38^ Habuchi et al. further combined the method with redox switching to see patterns as small as ∼80 nm.^39^ To distinguish phases in polymer–polymer mixtures, Kim et al. used spectrally resolved PAINT (SR-PAINT).^40^ The fluorescence of molecules adsorbed at the surface gives a different spectral behavior. The emission during localization is split into two channels: the localization channel and the spectra channel, which are further used to identify the phase.^41−43^ However, the total resolution, that is, the convolution of the localization density and the localization precision, suffers from the splitting of the light into two channels. Furthermore, the localization density must be relatively low to prevent overlap of the spectra of single molecules.

Here, we propose a new method that utilizes hydrogen bond-assisted adsorption to distinguish the phase difference in recycled PA/PE. We synthesized a new probe based on the well-known Nile Red stain: diOHNR has two additional hydroxyl groups at the aminoalkyl side chains (Figure 1A) and can be synthesized in two steps (Scheme S1). The two hydroxyl groups enhance the solubility of the probe in an aqueous solvent and prevent the aggregation that is typically observed in nonmodified Nile Red.^44^ Furthermore, the hydroxyl groups can promote the interaction between the PA and probe by forming hydrogen bonds, leading to a higher localization density and adsorption equilibrium at the PA phase than at the PE phase (Figure 1B). By comparing the localization density using a reference sample, such as pure PE and PA, the “chemical” phase structure can be resolved.

## Materials and Methods

### Materials

Chemicals and solvents for syntheses, purification, and analyses were purchased from Sigma-Aldrich, except naphthalene-1,3-diol (98%) that was obtained from Abcr GmbH. Ethanol used in the second step of the synthetic procedure was dried with 3 Å molecular sieves (Carl Roth GmbH). The probe, diOHNR, was synthesized in two steps, as described in the Supporting Information (Scheme S1). NMR spectra were obtained from solutions in DMSO-*d*_6_ and recorded by using a Bruker AV II 400 spectrometer. The spectra were analyzed with MestReNova.

### Photophysical Properties of diOHNR

Fluorescence spectra of diOHNR solutions, depicted in Figure S1, were recorded using a fluorescence spectrometer (SPEX Fluorolog 3-22 fluorimeter, Horiba). The quantum yields (ϕ_f_) of the molecule in various solutions were measured and calculated using the reference method described in the Supporting Information.^45,46^ These quantum yields are presented in Table S1. Fluorescence lifetimes (τ) of the probe were captured using a home-built time-correlated single-photon counting setup, detailed in a previous study.^47^ The instrument response function was determined by directly reflecting the laser light. The fluorescence lifetime data were fitted using the reconvolution method, implemented in FluoFit within Matlab (PicoQuant GmbH).^48^

The radiative decay rate (*k*_f_) of the molecule was determined using the relationship
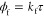
1

This rate was found to be 0.17 ± 0.03 ns^–1^. Utilizing the radiative decay rate, as demonstrated in Figure S2, we can deduce the fluorescence quantum yields of the probe at different polymer interfaces by measuring fluorescence lifetimes at various points along these interfaces.

### Sample Preparation

To test the feasibility of using the PAINT method to distinguish phase structures, a model system of polystyrene/poly(methyl methacrylate) (PS/PMMA), which has a well-known phase separation structure, was used.^49−52^ Polystyrene (M.W. 6,500,000, *M*_w_/*M*_n_ = 1.06) was purchased from Alfa Aeser GmbH, and poly(methyl methacrylate) (M.W. 7,000,000, *M*_w_/*M*_n_ = 1.1) was acquired from Agilent Technologies. The polymers were spin-coated onto a coverslip in a 1:4 weight ratio (PS/PMMA). Reference polyamide 6 (pellets), polyamide 66 (pellets), and polyethylene (low density, melt index: 25) were acquired from Sigma-Aldrich. A thin slice cut from a piece of recycled polyamide/polyethylene made by and extruding process was provided by BASF.^10^

### Fluorescence Microscopy and PAINT Analyses

**Figure 2:**
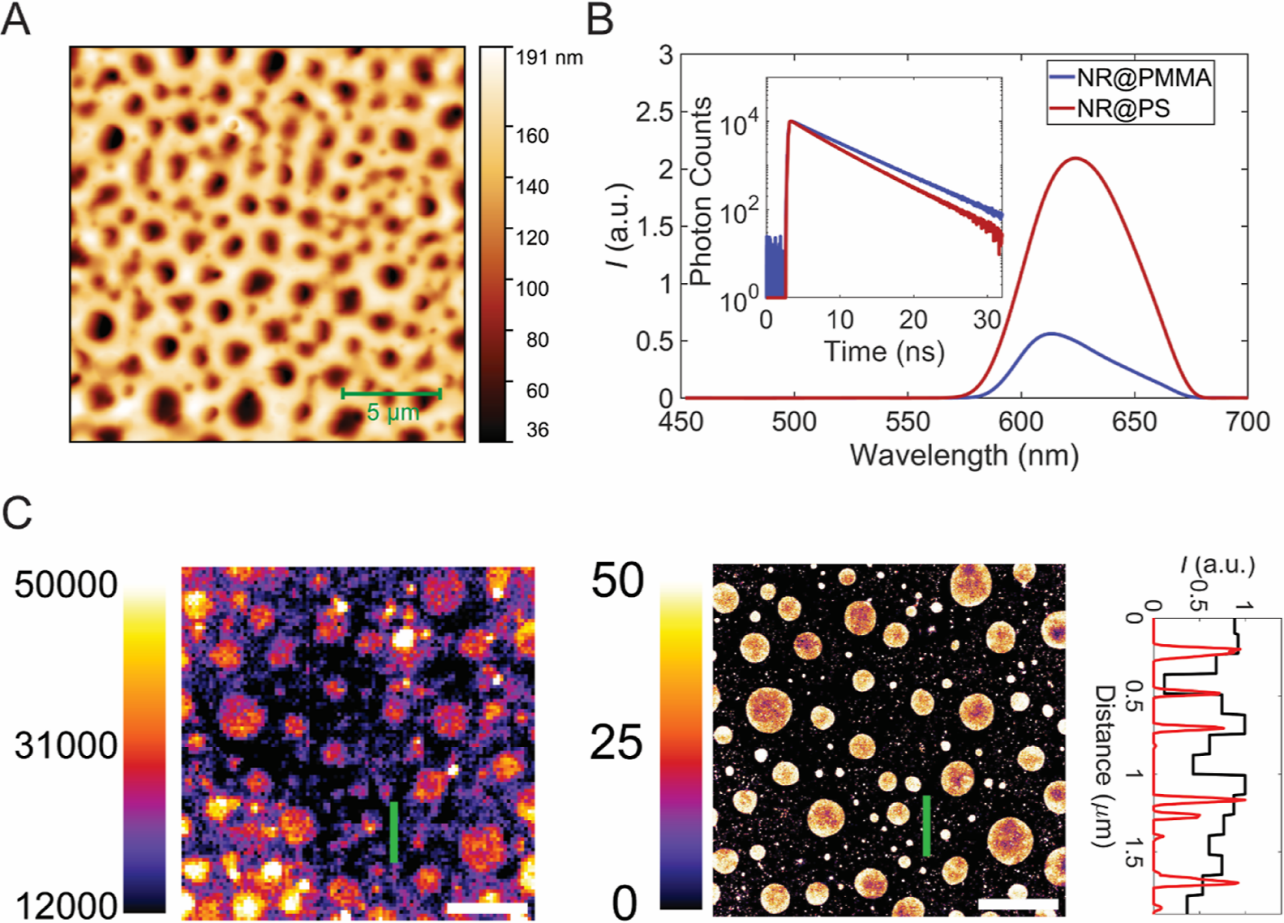
Feasibility check of the PAINT methodology using a layer consisting of PS and PMMA. (A) Height image obtained by AFM, revealing “island” structures characteristic of PS as previously reported.^49−52^ (B) Evaluation of the affinity of Nile Red for PS and PMMA using fluorescence spectra and time-correlated single-photon counting. By measuring the relative brightness, which is proportional to the product of Nile Red’s fluorescence quantum yield and its concentration at the surfaces, we calculated a ratio of *K*_PS_/*K*_PMMA_ = 4.3. (C) Diffraction limited fluorescence image (left) and PAINT image (right) of the same field. The PAINT image was reconstructed by applying known density filters to filter out localizations at the PMMA phase (Figure S3) and magnified 16 times (pixel size of 8.1 nm). The scale bar in the image represents 3 μm. The color scale represents camera intensity and photon counts for the diffraction limited image and the PAINT image, respectively.

Fluorescence images and super-resolution PAINT images in Figure 2 and 4 were acquired using a home-built total internal reflection microscope. The localization of the single emitters for PAINT images was performed using ThunderStorm,^53^ accompanied by the redundant cross-correlation implemented by Huang et al.^54^ The density filter was applied using the function in ThunderStorm based on the known average localization density of the probe at the reference samples, namely, pure PS, PMMA, PE, and PA polymers. The choice of the defined radii for the density filter depended on the pixel size of the reconstructed PAINT images.

**Figure 3:**
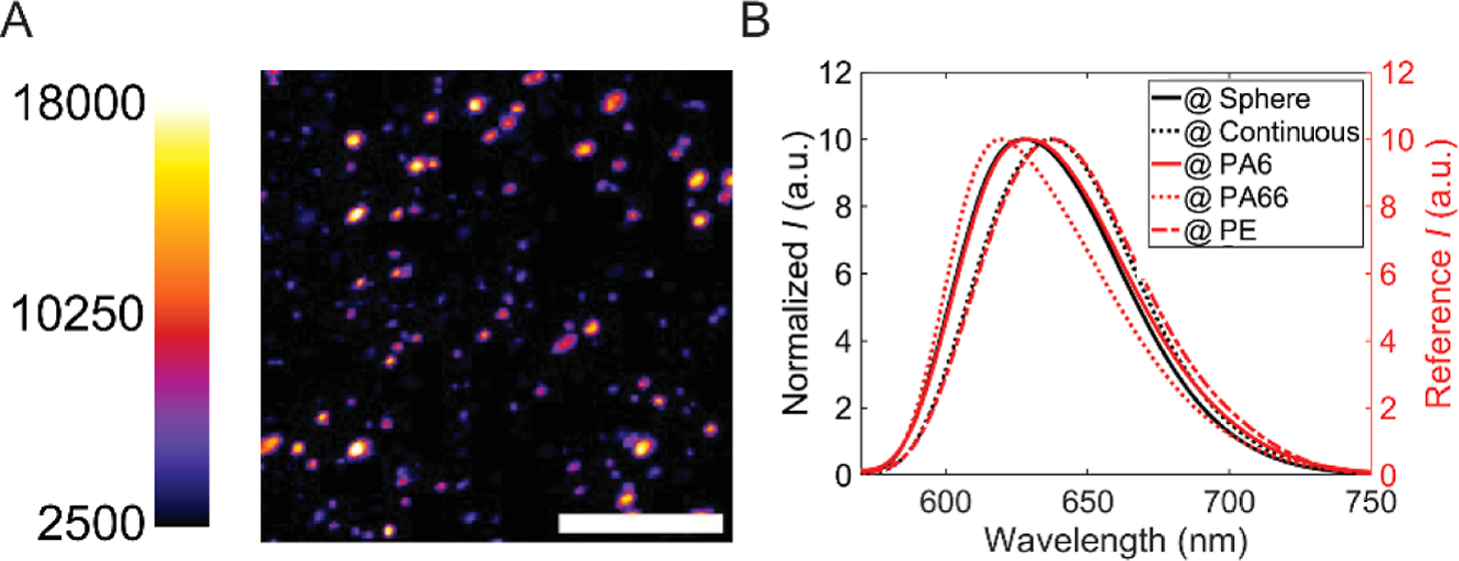
Screening of PA chemical structure using fluorescence imaging and spectra. (A) Fluorescence image of the recycled PA/PE measured by using 20 nM diOHNR with a confocal microscope, where spherical structures embedded inside the continuous phase are visible and the fluorescence intensity is higher in these structures. The scale bar is 10 μm, and the color scale represents photon counts. (B) Fluorescence spectra of diOHNR measured at the spherical phase (black line) and continuous phase (black dots) and compared to the spectra of reference samples, PA6 (red line), PA66 (red dots), and PE (red dash).

Fluorescence images in Figure 3 and the emission spectra of the probe at different polymer interfaces in Figures 2B and 3B were recorded with a confocal microscope (MicroTime 200, PicoQuant GmbH) extended with a spectrometer. The data were processed by using SymPhoTime64. Detailed experimental descriptions and parameters can be found in the Supporting Information.

## Results and Discussion

We measured the topography of the sample PS/PMMA (1/4 by weight ratio) using atomic force microscopy (AFM). Figure 2A shows the height profile of the sample, with PS “islands” ranging from 39 nm to 4 μm embedded inside the continuous phase of PMMA determined using the height of the domain, consistent with the known pattern of phase separation in this system. We used this layer to investigate whether we could recover the same type of phase structures using fluorescence microscopy. We first investigated the affinity of the nonmodified Nile Red probe toward different polymer surfaces by spin-coating pure PS and PMMA onto coverslips. The Nile Red probe was dissolved at a concentration of 20 nM in water to prevent aggregation, and the fluorescence spectra were measured using a spectrometer on the microscope. The results indicate that Nile Red has a higher affinity toward the PS phase. We calibrated the intensity using the fluorescence lifetime of each material (τ_PS_ = 3.43 ± 0.02 ns, τ_PMMA_ = 4.32 ± 0.02 ns) as a measure of the fluorescence quantum yield of the single molecule and obtained an equilibrium absorption ratio, *K*_PS_/*K*_PMMA_, of 4.3. We further used this value in PAINT imaging to determine the phases of the two distinct polymers.

We further quantified the total number of localization events of Nile Red (0.1 nM) at the reference PS and PMMA layers over 20 min (Figure S3). The event numbers show the same trend as that in Figure 2B, where the ratio of event numbers (PS/PMMA) is ∼4.3, which can thus be used as a filter to distinguish the chemical phase of the polymer. Next, we used a dilute Nile Red solution (0.1 nM) to perform the PAINT measurement on the PS/PMMA coating. The image reconstruction was done by applying a density filter of ∼25,000 events/μm^2^, that is, 30 events within a radius of 20 nm, to filter out the localizations at the PMMA phase. The density filter can be safely applied because the total event density is 4.3 times higher in PS than in PMMA (Figure S3). We directly compared the super-resolution image and the conventional image recorded at the same position (Figure 2C). The PS/PMMA phase was resolved in the reconstructed image, and the localization precision (σ_loc_) was ∼13.8 nm (Figure S4). Furthermore, we evaluated the total lateral resolution by Fourier ring correlation (FRC),^55^ that is, the convolution of the localization uncertainty and localization density, yielding σ_FRC_ = 18.8 nm (Figure S4). This indicates that the image resolution was faintly limited by the localization density typically observed in the SMLM images. In Figure 2C, we plotted the intensity profile of the line indicated in the images. With the conventional image, the nanoscopic structures were not resolved and could be overestimated in size. In contrast, the PAINT method allowed us to recover most of the nanoscale phase separation domains.

We proceeded to measure the phase structures of the recycled PA/PE (20/80 wt). However, unlike the model system, where the chemical structures of the two polymers are known, the exact chemical phase of the recycled PA material is unknown on a nanometer scale. To address this, we utilized a relatively concentrated probe solution (20 nM of diOHNR in ethanol) to stain the surface of the recycled material (Figure 3A). The fluorescence image exhibited contrast, revealing the presence of nanoscopic-to-microscopic spherical structures embedded in the continuous phase. We then closely measured the fluorescence spectra at these two distinct regimes and compared them to the spectra of diOHNR staining common PA materials such as polyamide 6 (PA6) and polyamide 66 (PA66). In Figure 3B, the spectrum recorded at the spherical structure was found to be identical to that measured at the PA6 surface, which indeed is the chemical structure of the recycled PA material. Moreover, the spectrum observed at the continuous phase closely resembled that measured at PE interfaces. Therefore, it is evident that the polymer is composed of spherical PA6 structures embedded within a PE matrix, as also found by other studies on similar PE/PA blends.^9,17,56^

**Figure 4:**
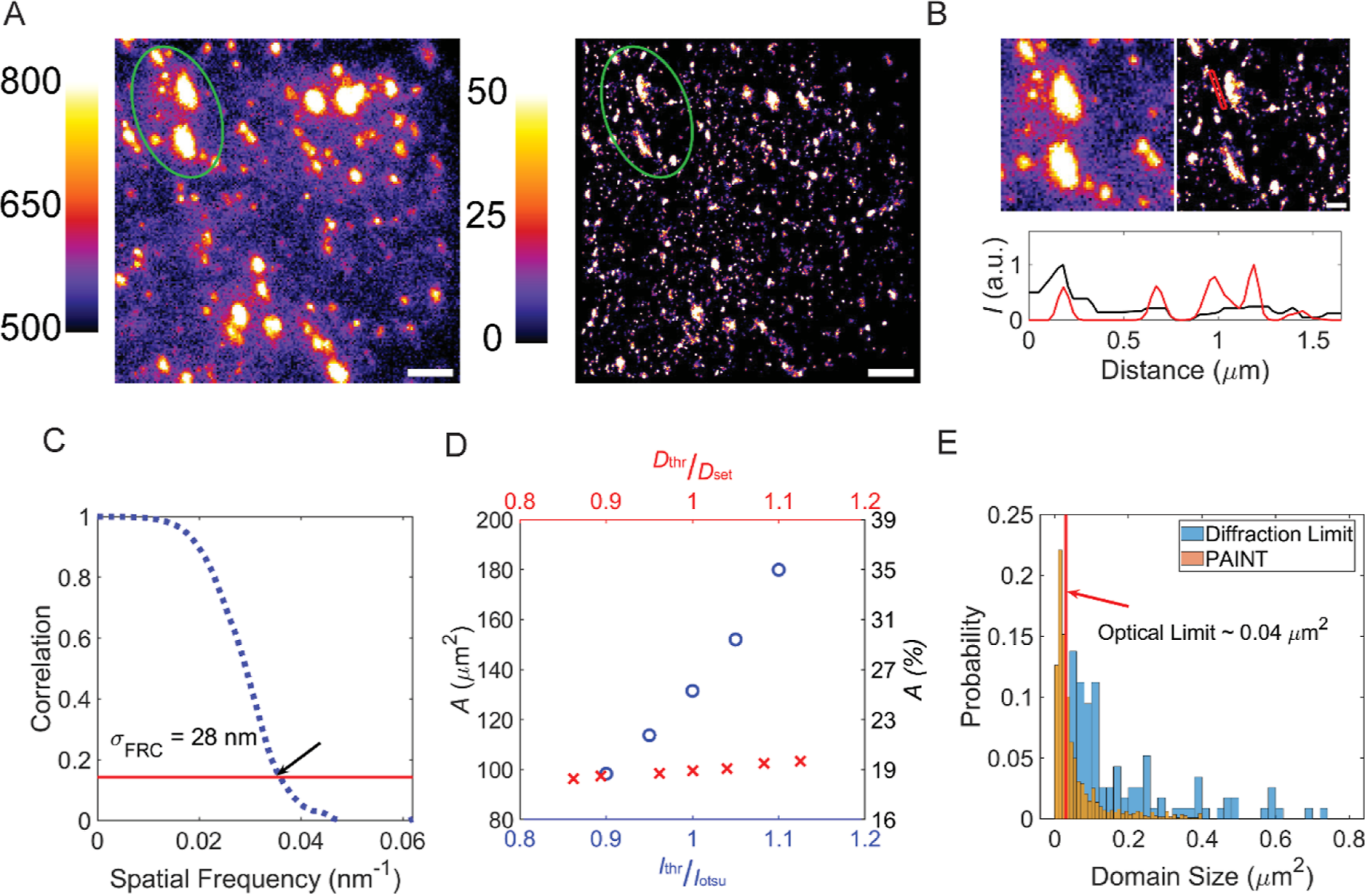
Super-resolution images of recycled PA/PE. (A) Left: diffraction limited fluorescence image, using diOHNR (20 nM) to stain [color bar indicates intensity (au)], Right: PAINT image, using 0.3 nM diOHNR (color bar indicates photon counts); scale bar represents 3 μm. The image was reconstructed by applying a density filter as indicated in Figure S5, and the image was magnified by 5 times (pixel size = 25.8 nm). The bright spot represents PA. The color bar represents camera intensity for the diffraction limited image, and it denotes photon counts for the PAINT image. (B) Enlargement of the regime indicated by the green circle in (A); the scale bar represents 1 μm. The plot below shows the line profile along the red line in the image. (C) The conclusion from the plot is that the lateral resolution of the PAINT image from FRC calculation is equal to 28 nm. (D) PA areas *A* (in μm^2^ and %) as found by the two methods as a function of the threshold intensity *I*_thr_ divided by the Otsu threshold value *I*_otsu_. *D*_thr_ and *D*_set_ are the threshold density and set density in Figure S5, which are used to calculate the PA area in the PAINT image. (E) Size distribution of the PA particles obtained from the conventional (orange) and PAINT (blue) methods. With the PAINT method, we found that approximately 60% of PA domains have sizes below the diffraction limit. The average size is 0.088 μm^2^.

To distinguish the phase structure of the recycled material on the nanometer scale, we measured the localization density of diOHNR (0.3 nM) at the reference polymers, that is, pure PA6 and pure PE. The difference in localization densities at PA and PE was about 13 times (Figure S5), which allowed us to measure the super-resolved phase structure of the PA/PE. The fluorescence quantum yields of diOHNR in PA and PE derived from the lifetimes (Figure S6) and radiative rate constant (Figure S2) are determined to be 0.71 ± 0.11 and 0.59 ± 0.10, respectively. These findings suggest that the influence of the quantum yield on localization densities is relatively subtle. Furthermore, using the nonmodified NR to perform the same localization comparison did not yield a significant contrast, that is, density difference, at the two distinct phases (Figure S7). Thus, the modification of NR with the hydroxy groups is crucial for the success of the PAINT imaging of PA/PE. In Figure 4A, we reconstructed the PAINT image by applying a density filter (∼470 events/μm^2^, i.e., 4 events within a radius of 50 nm) after the localization process. We further recorded the conventional image on the same area, but with a higher concentration of diOHNR for staining the surface (20 nM). The PAINT image provided more structural details, allowing us to zoom in on the area indicated by the green circle in Figure 4A. In this area, we observed many nanoscopic structures below the diffraction limit, with sizes ranging from ∼200 to ∼50 nm. A line profile across the area (red line in Figure 4B) reveals many nanoscopic domains. We further used FRC to confirm the total lateral resolution, which was found to be 28 nm, demonstrating that the domains are not the result of noise.

With the PAINT method, we can better characterize the total area of the PA domains and the average size of the structures. In Figure 4D, we plot the total area of the PA domains as obtained from the two methods. The area defined by the conventional method was determined using the Otsu thresholding method, which distinguishes the foreground and background by minimizing intraclass intensity variance.^57−59^ The area defined by the PAINT method was determined by counting the number of nonzero pixels. This is due to the nature of the image reconstruction process, which filters out the localization at PE, so the PE area should represent zero in the postprocessed image. The areas determined by the two methods are clearly different, with the conventional method showing a surface area that contains 27 ± 6% PA, compared to 19 ± 3% with the PAINT method. We argue that the sizes of the areas determined by the conventional diffraction limited staining method are overestimated, as shown in Figure S8. The signal-to-noise ratio in Figure 4A is low, resulting in the counting of some undefined areas as PA areas, while some of the PA particles are filtered out during the thresholding process because their size is too small and their fluorescence intensity is relatively low as it is distributed over the diffraction limited spot. We further estimated the reliability of the area defined by the two methods by varying the threshold value, that is, the threshold intensity and the density filter. We adjusted the thresholding value by ±10% for both methods. The area defined by the conventional method is highly sensitive to the threshold, with defined areas varying by approximately 80%. This is not the case with the super-resolution method, in which the area only changes by approximately 5% from *D*_thr_/*D*_set_ = 0.9–1.1, and the ratio of PA/PE is close to the known bulk mixing ratio. The PAINT method detects molecules on the surface. However, the surface structure is likely to reflect the bulk mixture due to the isotropic nature of the material.

Finally, we compared the size distribution of the PA domains obtained from the two methods, as shown in Figure 4E. For the conventional method, the number of PA particles is around 600, and all of them have a size above the diffraction limit, that is, 0.04 μm^2^. However, when using the PAINT method, we found that approximately 60% of the PA particles have a size below the diffraction limit. Furthermore, the number of domains is twice more than the number found by the conventional method. These two results indicate that most of the PA structures are on the nanoscale. Thus, they can be overlooked by the diffraction limited imaging method. Some of the PA6 domains might still be slightly overestimated due to the limited resolution of the PAINT method, which operates at a resolution in the tens of nanometers range. When compared to the current “standard” imaging methods such as SEM or TEM, SMLM exhibits a resolution that is an order of magnitude lower. Nonetheless, this approach effectively characterizes the recycled material. This effectiveness stems from the observation that the peak probability of the PA size falls within the range of 0.01–0.02 μm^2^, equivalent to 100–141 nm in length values that exceed the resolution threshold of our method. Furthermore, in traditional electron-microscopy-based techniques, it is essential to maintain a sample thickness below 100 nm. Consequently, during the slicing process, there is a possibility of PA domain detachment, which can introduce a degree of bias into the results.^9,17^

## Conclusions

In conclusion, we proposed a new method to visualize the “chemical phase structure” of the recycled PA/PE by exploiting the hydrogen-bond-assisted interaction. Our newly synthesized probe, diOHNR, performed better than the commonly used Nile Red, as it demonstrated improved solubility and higher localization density at the polyamide phase. By measurement of the localization density with the reference polymers, the appropriate density filter can be utilized to distinguish the phases quantitatively. The spectral behavior of the probe was used to screen the microscopic phase structure, which was confirmed as Polyamide 6 particles embedded in a continuous PE phase. On a reference sample, the localization density of diOHNR at Polyamide 6 was 13 times higher than the one measured at PE; this allowed us to render the phase structure of recycled PA/PE. Our results showed that over 60% of the PA particles were smaller than the optical limit, which provides valuable insights into the size distribution to the mechanical properties of the recycled materials. The PAINT technique holds significant promise for the recycling industry as it can help to enhance the quality of recycled plastics. By providing insight into the sizes of the polymer phases within the recycled stream, this method could aid in the development of more effective additives for recycling. Moreover, it has the power to fundamentally shift the recyclability perspective of materials that are presently deemed nonrecyclable.

## Data Availability

Data and scripts to reproduce all the figures can be found at Figshare: doi.org/10.21942/uva.23580459.

## Supporting Information Available

Materials and methods; synthetic procedure of diOHNR; fluorescence spectra and time-correlated single-photon counting curves of diOHNR in different solvents; fluorescence quantum yield versus fluorescence lifetime; localization density of 0.1 nM Nile Red at pure PS and PMMA sample; histogram of localization uncertainty; fluorescence decays of diOHNR; ^1^H NMR, ^13^C NMR spectrum of diOHNR; and photophysical properties of diOHNR in various solvents (PDF)
Raw single molecule images of diOHNR at PA/PE recycled material (AVI)

## Supplementary Material

la3c01976_si_001.pdf

la3c01976_si_002.avi
