# Supplementary material for: Super-resolution Fluorescence Imaging of Recycled Polymer Blends via Hydrogen Bond-Assisted Adsorption of a Nile Red Derivative
Source: Langmuir. 2023 Oct 3;39(41):14652–9. doi: 10.1021/acs.langmuir.3c01976 (PMC10586370; doi:10.1021/acs.langmuir.3c01976)
Supplement: Supplementary file 1 — la3c01976_si_001.pdf [file la3c01976_si_001.pdf]

SUPPORTING INFORMATION

Super-resolution Fluorescence Imaging of Recycled Polymer Blends via Hydrogen Bond Assisted Adsorption of a Nile Red Derivative

Chao-Chun Hsu,[1*] Markus Rückel,[2] Daniel Bonn,[3] Albert M. Brouwer[1]

[1] van 't Hoff Institute for Molecular Sciences, University of Amsterdam, Science Park 904, 1098 XH Amsterdam, The Netherlands

[2] Group Research, BASF SE, Ludwigshafen D-67056, Germany

[3] van der Waals-Zeeman Institute, Institute of Physics, University of Amsterdam, Science Park 904, 1098 XH Amsterdam, The Netherlands

Email: c.c.hsu@uva.nl (C.C. Hsu) a.m.brouwer@uva.nl (A.M. Brouwer)

SUPPORTING INFORMATION

Table of Contents

Materials and Methods....................3
Supplementary Figures and Tables....................4
References....................10

Materials and Methods

In Scheme S1 we show the synthetic procedure for 9-(bis(2-hydroxyethyl)amino)-5H-benzo[a]phenoxazin-5-one (diOHNR). To synthesize 2,2'-((4-nitrosophenyl)azanediyl)bis(ethan-1-ol) (a), 9.06 g (50 mmol) of N-phenyldiethanolamine is suspended in 22 mL 37% HCl at 5 ºC for 30 mins. Sodium nitrite (6.67g, 97 mmol) is dissolved in 10 mL water, and the solution was added dropwise to the reaction flask using a syringe pump over 40 mins to prevent formation of NO_2 gas. After the addition of $NaNO_2$, the mixture was stirred for another 2 hrs. The reaction mixture turns into a slurry, and it is filtered to remove water. The solid phase was washed with several portions of ethanol and acetone to give 7.37 g of the yellow nitroso-compound.

To synthesize diOHNR, the nitroso-compound (3.15 g, 15 mmol) was added portion-wise to a refluxing solution of naphthalene-1,3-diol (2.0 g, 12.5 mmol) in 65 mL of ethanol over 45 mins. The reaction was continued for another 3.5 hrs. Afterwards, the solvent was evaporated, and diOHNR was purified by column chromatography using ethyl acetate followed by methanol/dichloromethane (1/10) to afford pure diOHNR (300 mg, 7 %). ^{1}H NMR (400 MHz, DMSO) δ 8.56 (d, J = 8.0 Hz, 1H), 8.13 (dd, J = 7.8, 1.4 Hz, 1H), 7.82 (td, J = 7.6, 1.6 Hz, 1H), 7.77 – 7.68 (m, 1H), 7.62 (d, J = 9.1 Hz, 1H), 6.90 (dd, J = 9.1, 2.7 Hz, 1H), 6.76 (d, J = 2.6 Hz, 1H), 6.31 (s, 1H), 4.95 – 4.83 (m, 2H), 3.62 (q, J = 4.9 Hz, 8H). ^{13}C NMR (101 MHz, DMSO) δ 182.46, 152.41, 152.32, 146.62, 138.95, 132.10, 132.04, 131.54, 131.09, 130.51, 125.52, 124.82, 123.85, 111.12, 105.07, 97.02, 58.65, 53.90. ^{1}H and ^{13}C NMR spectra are shown in Figure S6 and S7. m/z: [M + H]+ calcd for $C_{20}H_{18}N_2O_4$ 351.1345; Found 351.1339

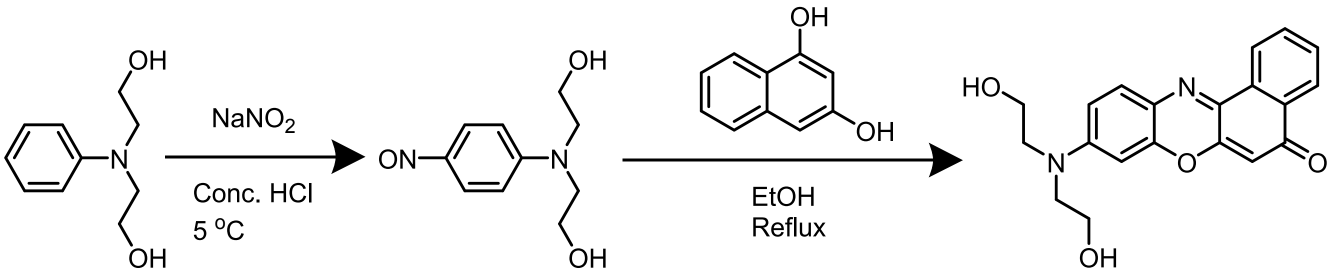

Scheme S1. Synthetic procedure of diOHNR.

The fluorescence quantum yield was determined using the equation:

$$\phi_{f,x} = \phi_{f,st}\frac{F_x f_{st}}{F_{st} f_x}\left(\frac{n_x}{n_{st}}\right)^2 \tag{1}$$

Here $\phi_{f,x}$ is the fluorescence quantum yield of the sample, $\phi_{f,st}$ denotes the quantum yield of the standard molecule. *F* stands for the integrated emission, and *f* represents the absorption factor. *n* is the refractive index of the solvent. During the quantum yield measurement, Rhodamine 6G (Sigma-Aldrich) in ethanol ($\phi_{f,st} = 0.91$) was used as the standard for calculating the quantum yield of diOHNR in dioxane and dichloromethane. Rhodamine 101 (Sigma-Aldrich) in ethanol ($\phi_{f,st} = 0.915$) was used as reference for diOHNR in ethanol and water.[1]

Samples of reference PS, reference PMMA, and PS/PMMA (1/4) were spin coated from 3% wt polymer in toluene onto a 22×22 mm^2 high-precision coverslips (Paul Marienfeld GmbH, 170 µm). The spinning rate was 2000 rpm for 60 s. After coating, the samples were dried in a vacuum oven for 3 hours at 40°C to evaporate all residual solvent. Reference PE was dissolved in decaline (3% wt) while reference PA was dissolved in trifluoroacetic acid/acetone (3/1). A 10 µm thick slice of recycled PA/PE was kindly provided by BASF, and the sample was attached to the coverslips by annealing in the oven at 120°C for 10 minutes.

The probe solution was prepared immediately before measurement by diluting a 1 µM stock solution of Nile Red (for PS/PMMA) or diOHNR (for PA/PE) in ethanol to the desired concentration with MilliQ water. The coverslips with the coating or recycled material were placed on the microscope. A hollow tube was placed on top of the coverslip to hold the solution containing the probe. Fluorescence lifetimes and conventional fluorescence images

from Figure 3A were measured using a MicroTime 200 confocal microscope (PicoQuant GmbH) with an Olympus IX-71 microscope body and a 100×1.4 N.A. objective (UplanSApo, Olympus). Excitation light at 561 nm was generated using an NKT Supercontinuum Laser (SuperK Extreme Supercontinuum, NKT Photonics), and emission from the sample was detected using time-resolved single photon counting (TCSPC) with a PDM Series detector (PicoQuant GmbH). Decay time traces were processed and fitted with SymPhoTime64 using the Tailfit method. Steady-state emission spectra were measured using an EMCCD (PhotonMAX, Princeton Instruments/Acton) camera attached to a spectrometer (Spectra Pro-150, Acton Research Instruments). The wavelength of the spectrograph was calibrated by a 2nd order polynomial using the NKT Supercontinuum Laser at 540 nm, 560 nm, and 580 nm.

PAINT images and diffraction limit images from Figure 2A and Figure 4 were recorded with a home-built widefield/TIRFM microscope, and excitation at 561 nm was generated using a continuous wave laser (Cobolt 06-DPL, HÜBNER Photonics GmbH).[2] The excitation laser's beam diameter was 5 mm and was expanded five times by passing through a telescope and the center part was selected using an iris. The laser was reflected by a 488/560 nm dichroic mirror and focused by a positive lens right before the objective (UPLAPO100XOHR, Olympus). After passing through an 593LP filter and a 561 nm notch filter, the sample's emission was collected by an sCMOS camera (Orca-Flash4.0 V2, Hamamatsu Photonics). The acquisition rate was set at 15 ms/frame for PS/PMMA samples and 50 ms/frame for PA/PE samples.

Super-resolution images were analyzed using the *ThunderStorm* plugin[3], and all analyses were performed after drift correction in Matlab using the redundant cross-correlation algorithm by *Huang et al*.[4] In a typical routine, we first measured the reference samples and determined the localization density at each reference sample by measuring the total localization events over the excitation field in 20 minutes (see Figure S1 and Figure S3). By dividing the event numbers by the area, we obtained the localization density at different reference samples. We measured the polymer mixtures, i.e., the PS/PMMA and PA/PE samples, using the same parameters as for the reference sample. After applying the cross-correlation procedure, we used the density filter implemented in *ThunderStorm* to filter out the localization events at the phase with less localization density. For the PS/PMMA system, the localization density is lower in PMMA, so we filtered it out during the density filtering, which we set at 24000 events/μm^2 (i.e., 30 events in a radius of 20 nm). For recycled PA/PE, PE has 13 times less localization event density than the PA phase, so we set the filter at 470 events/μm^2 to filter out the localization at the PE phase. The large difference in density could be attributed to different probes used or to the solution used for the measurement. In the model system, we used water because ethanol would dissolve PMMA, while in the PA/PE system, we used ethanol. After filtering, we reconstructed the images using the default average-shifted method.

The topography of the coating was recorded using an Atomic Force Microscope (AFM) (Nanowizard 3, JPK Instruments). Images were acquired using tapping mode with an Al-coated silicon tip (Nanoworld Point probe silicon SPM sensor, resonant frequency~320kHz). The pixel size of the image is 39 nm. The images were analyzed using Gwyddion.[5]

Supplementary Figures and Tables

Figure S1 and Table S1 present a summary of the photophysical properties of diOHNR in various solvents. Figure S2 illustrates that the radiative decay rate of the molecules can be determined by fitting a linear curve of quantum yield against fluorescence lifetime.

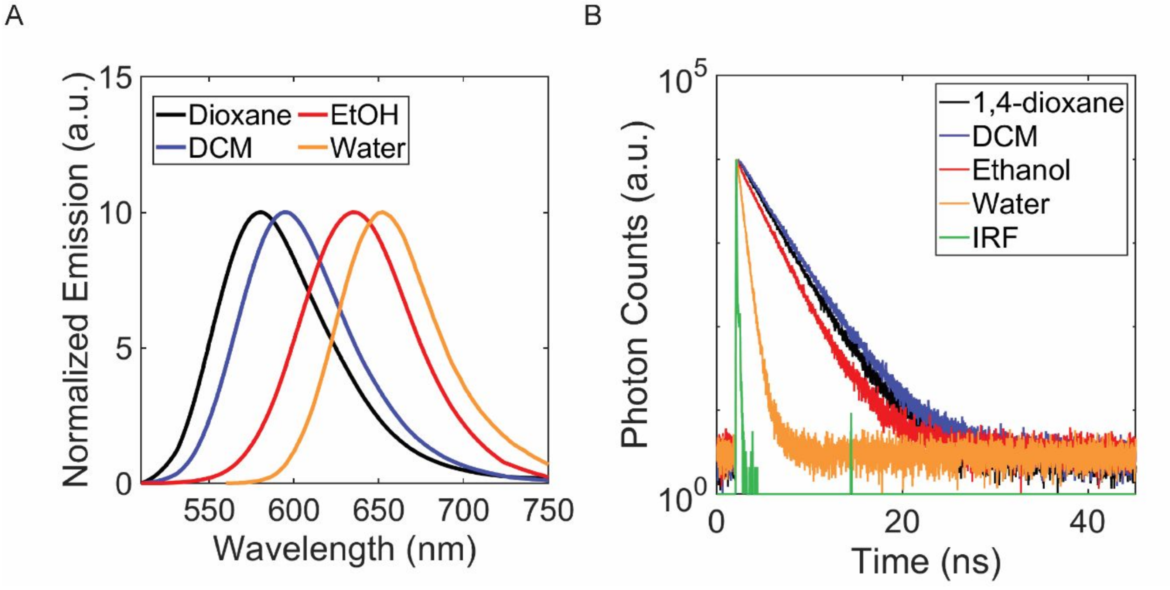

Figure S1 (A) Fluorescence spectra and (B) time-correlated single-photon counting curves of diOHNR in different solvents. "IRF" refers to the instrument response function, and "DCM" and "EtOH" represent dichloromethane and ethanol, respectively**.**

Table S1 Photophysical properties of diOHNR in different solvents

	ϕ_f	τ (ns)
Dioxane	0.75	4.07
DCM	0.69	4.28
EtOH	0.48	3.17
Water	0.08	0.85

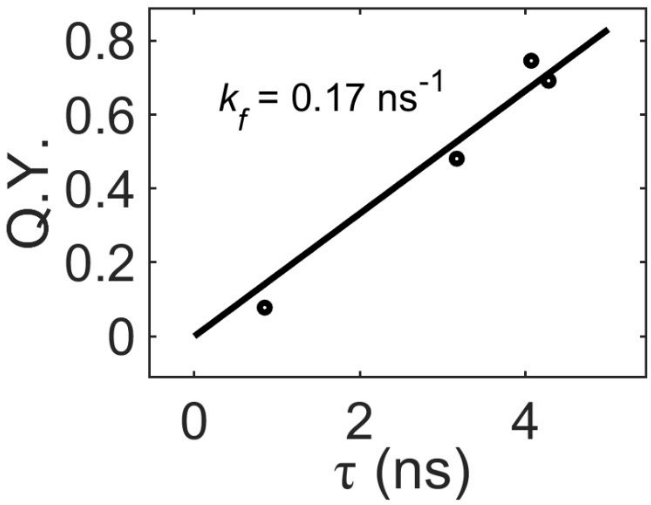

Figure S2 Fluorescence quantum yield versus fluorescence lifetime. The data points were fitted with a linear curve, and the slope represents the radiative dacay rate.

Figure S3 shows the localization density of Nile Red at the reference PS and PMMA coatings, which were measured over 20 minutes using a 0.1 nM solution.

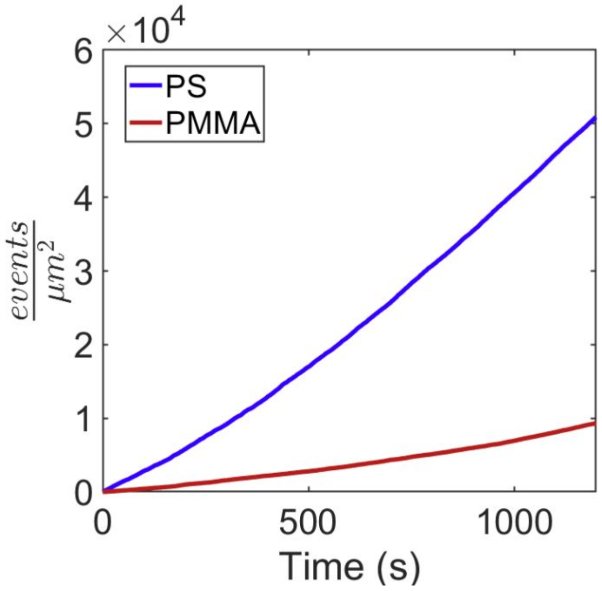

Figure S3 Localization density of 0.1 nM Nile Red at pure PS and PMMA sample. The event density at PS over 20 mins is ~50000 while that on PMMA is ~10000. The density filter in Figure 2D was chosen at ~24000 which guarantees to filter out all the localization events at the PMMA phase.

Figure S4 shows average the localization precision (σ_{loc}) and the total resolution, σ_{FRC}, determined by using the Fourier Ring Correlation method.[6]

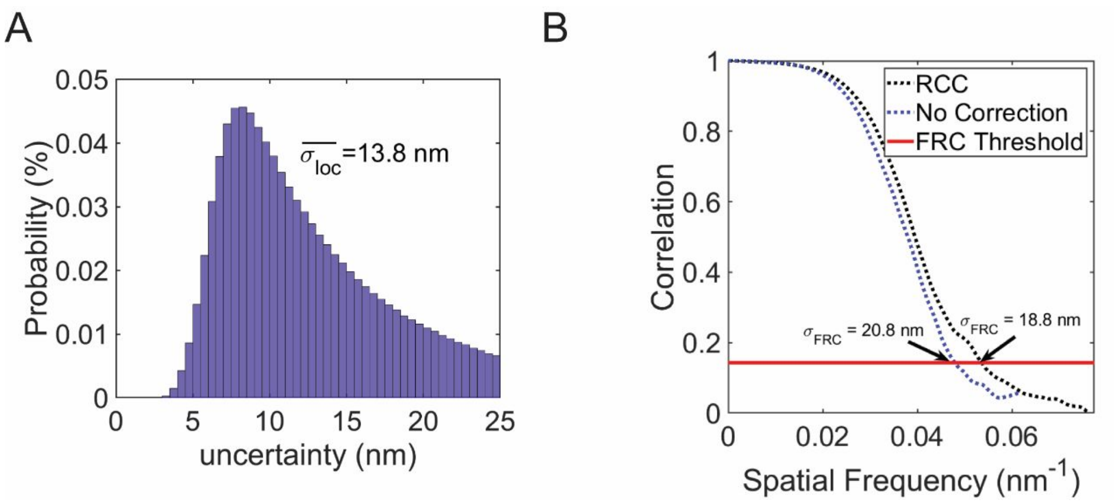

Figure S4 (A) The histogram of localization uncertainty. The average $\boldsymbol{\sigma_{loc}}$ is equal to 13.8 nm. (B) The $\boldsymbol{\sigma_{FRC}}$ of the PS/PMMA (1/4) showing in Figure 2C. The $\boldsymbol{\sigma_{FRC}}$ after the redundant cross-correlation processing gives an improved resolution ~18.8 nm.

Figure S5 demonstrates the localization density measured at the reference PA6 and PE coating using diOHNR. The dye concentration is 0.3 nM, and the localization event numbers were measured over 20 mins.

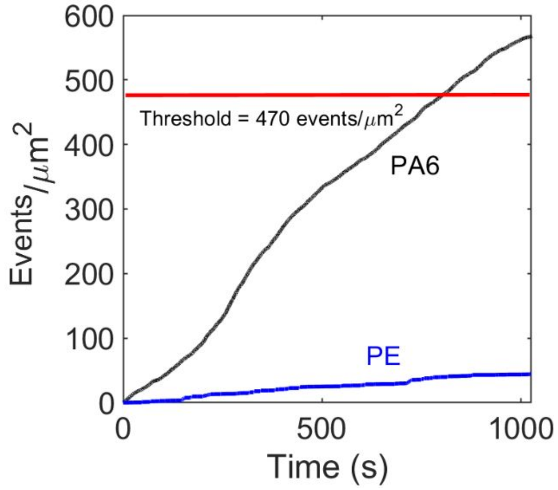

Figure S5 Localization density of 0.3 nM diOHNR at pure PA6 and PE samples. The event density at PA6 over 20 mins is ~580 while the density at PE is ~45. The density filter in Figure 4 was chosen as 470 events/μm^2 which guarantees to filter out all the localization events at the PE phase. We also evaluated whether the density filter affects the results, i.e. the total area of PA6, average particle size of PA6. The area was found to vary <5% with filter changing over 20%.

In Figure S6, we show the time-resolved fluorescence of diOHNR at PA6 and PE. The flurescence lifetimes of the prove at PA6 and PE are 4.25 ± 0.02 ns and 3.56 ± 0.05 ns.

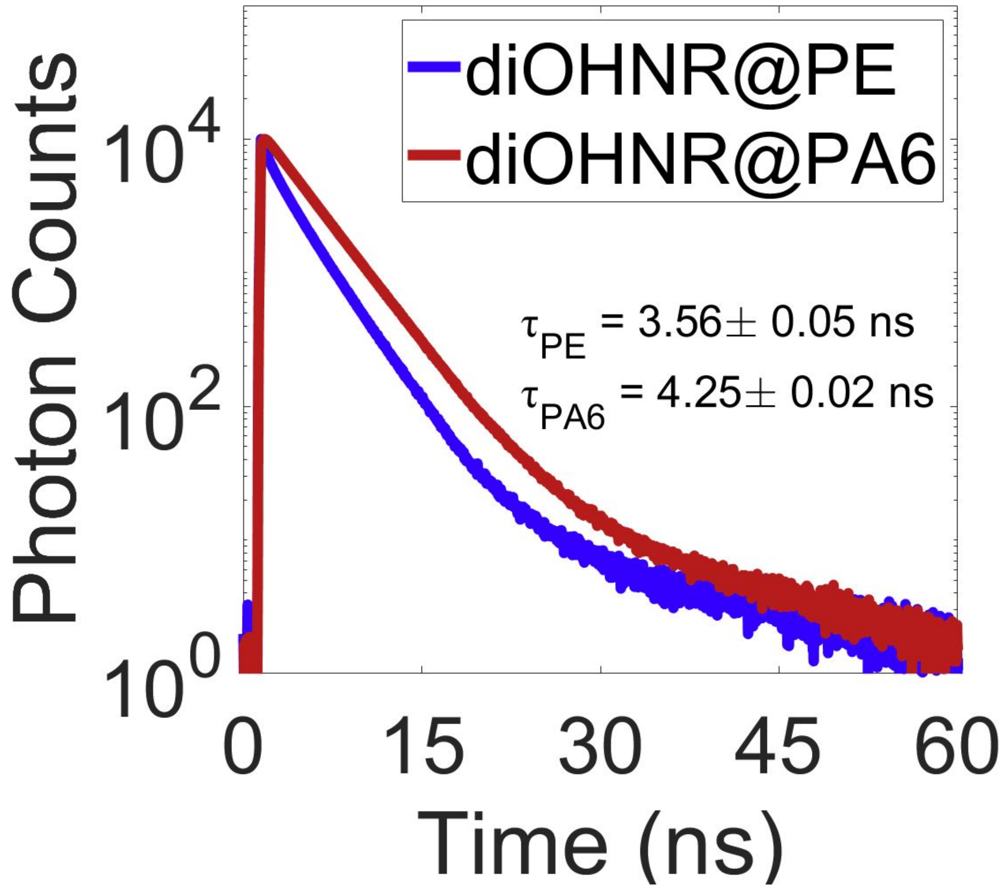

Figure S6 Fluorescence decays (TCSPC) of diOHNR at different reference polymers.

In Figure S7, we tested whether we can resolve a similar PA/PE phase separation structure using commercially available Nile Red. We found there is almost no contrast: Nile Red has similar localization density at PE and PA6, and, thus, no pattern was resolved.

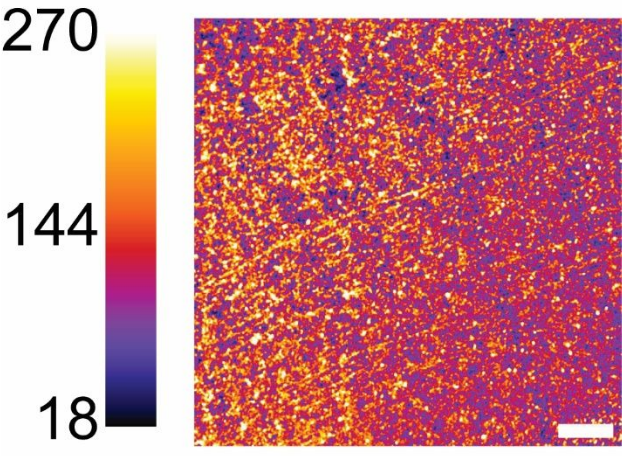

Figure S7 The PAINT image of recycled PA/PE measured using commercially available Nile Red. There is no preference of the “normal” Nile Red over PA and PE; thus, no pattern was resolved. Scale bar = 3 μm.

In Figure S8, we demonstrate the PA area resolved by the diffraction limited method using the TIRF microscope. The fluorescence image was binarized by the Otsu thresholding method, and the foreground (black) is the PA phase, which possesses higher intensity. The image is very fuzzy and the area is sensitive to the threshold. We varied the threshold value by 20% (Figure 4D), and the total PA area defined thresholding is changing by 80%.

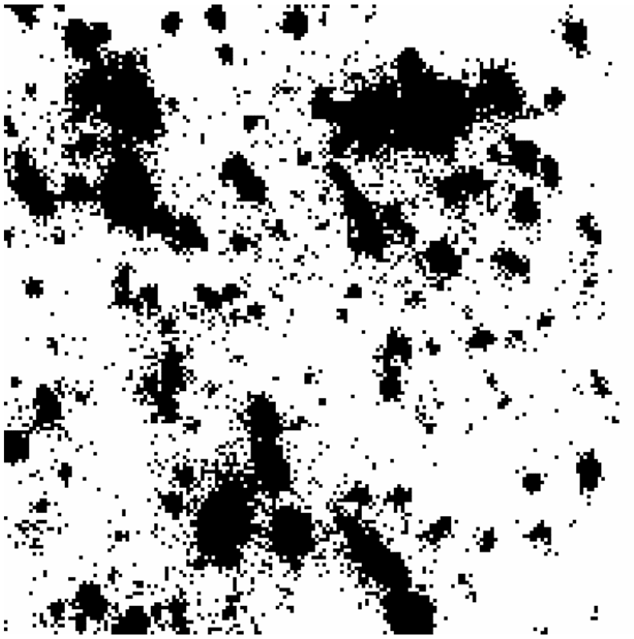

Figure S8 The binarized image of recycled PA/PE measured by conventional method. Since diOHNR has higher preference over PA phase, it will have higher localization and fluorescence intensity. This gives a foreground and background which we used Otsu threshold to define the area of PA.

In Figure S9, S10, and S11, we show the ^{1}H, ^{13}C NMR, and mass spectra of the newly synthesized probe, diOHNR.

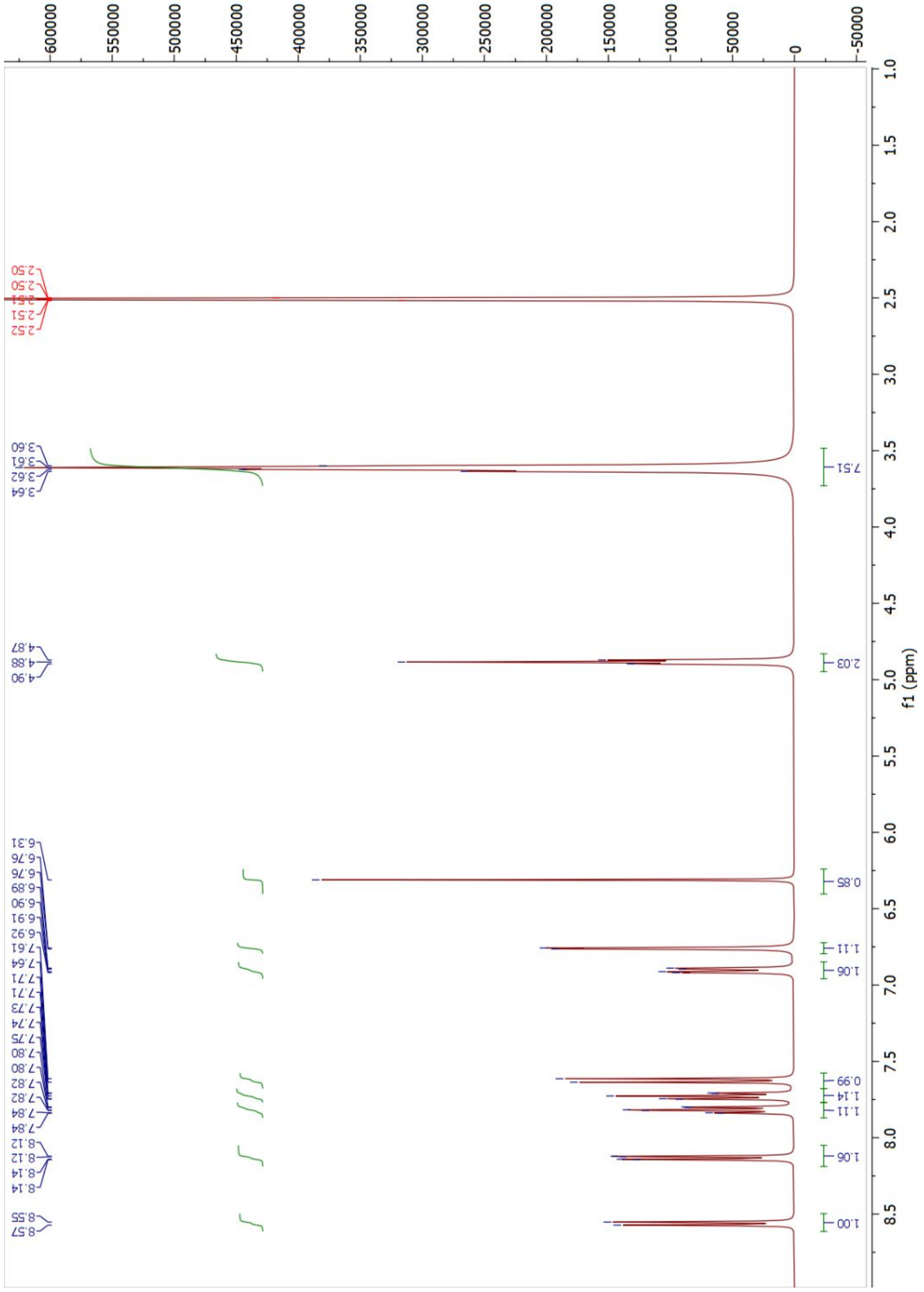

Figure S9 ^{1}H NMR spectrum of diOHNR. Solvent: DMSO.

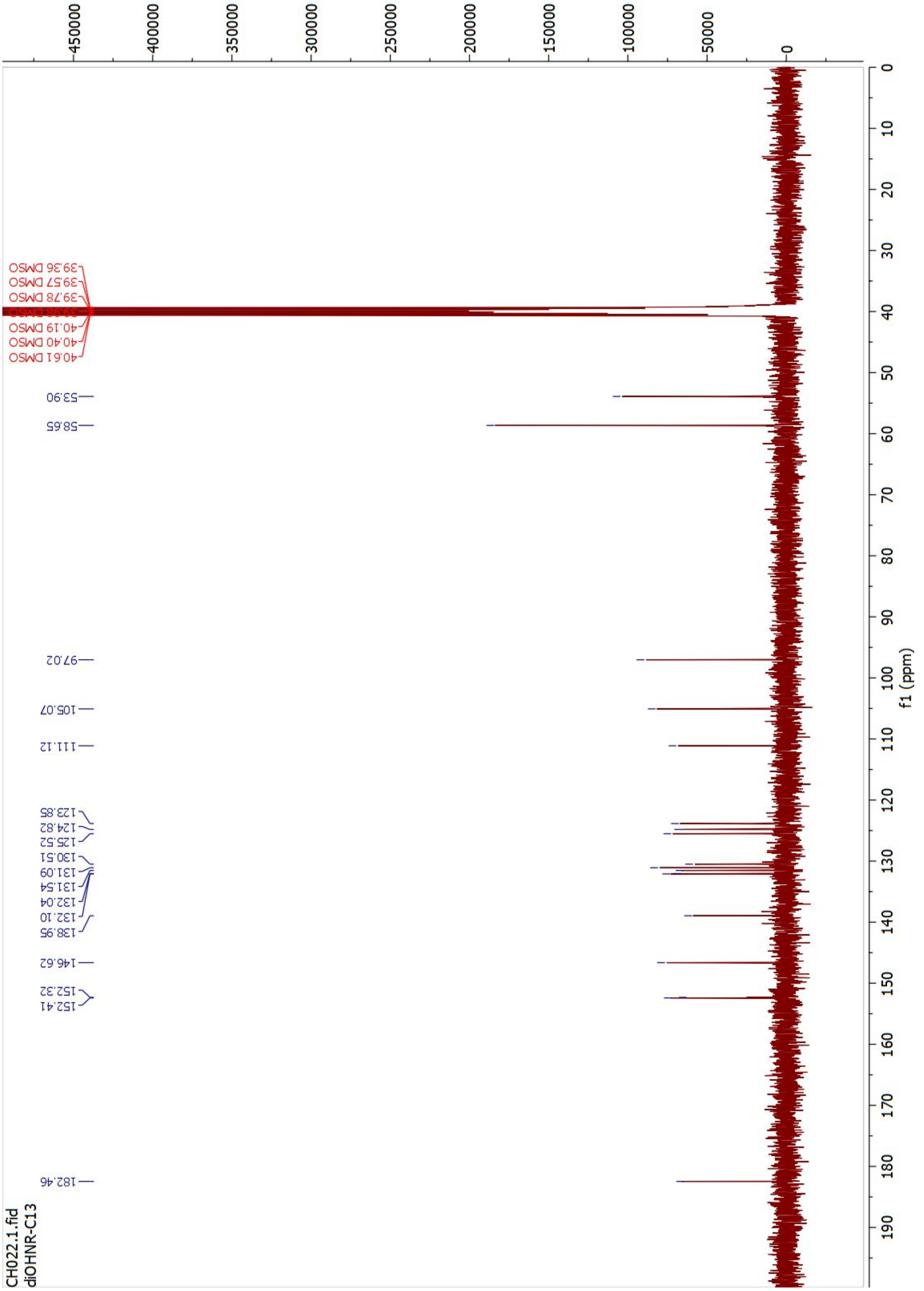

Figure S10 ^{13}C NMR spectrum of diOHNR.Solvent: DMSO

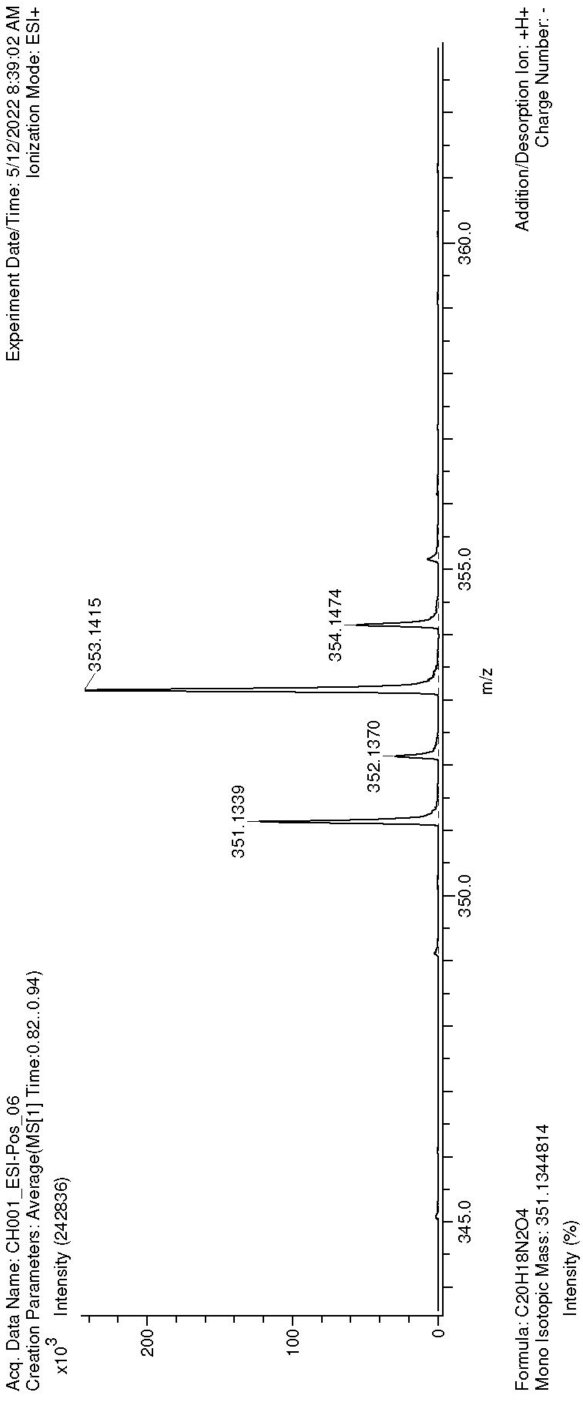

Figure S11 [13]Mass spectrum of diOHNR.

References

(1) Würth, C.; Grabolle, M.; Pauli, J.; Spieles, M.; Resch-Genger, U. Relative and Absolute Determination of Fluorescence Quantum Yields of Transparent Samples. *Nat Protoc* **2013**, *8* (8), 1535–1550. https://doi.org/10.1038/nprot.2013.087.
(2) Hsu, C.-C.; Hsia, F.-C.; Weber, B.; de Rooij, M. B.; Bonn, D.; Brouwer, A. M. Local Shearing Force Measurement during Frictional Sliding Using Fluorogenic Mechanophores. *J. Phys. Chem. Lett.* **2022**, *13* (38), 8840–8844. https://doi.org/10.1021/acs.jpclett.2c02010.
(3) Ovesný, M.; Křížek, P.; Borkovec, J.; Švindrych, Z.; Hagen, G. M. ThunderSTORM: A Comprehensive ImageJ Plug-in for PALM and STORM Data Analysis and Super-Resolution Imaging. *Bioinformatics* **2014**, *30* (16), 2389–2390. https://doi.org/10.1093/bioinformatics/btu202.
(4) Wang, Y.; Schnitzbauer, J.; Hu, Z.; Li, X.; Cheng, Y.; Huang, Z.-L.; Huang, B. Localization Events-Based Sample Drift Correction for Localization Microscopy with Redundant Cross-Correlation Algorithm. *Optics Express* **2014**, *22* (13), 15982. https://doi.org/10.1364/OE.22.015982.
(5) Nečas, D.; Klapetek, P. Gwyddion: An Open-Source Software for SPM Data Analysis. *Open Physics* **2012**, *10* (1). https://doi.org/10.2478/s11534-011-0096-2.
(6) Nieuwenhuizen, R. P. J.; Lidke, K. A.; Bates, M.; Puig, D. L.; Grünwald, D.; Stallinga, S.; Rieger, B. Measuring Image Resolution in Optical Nanoscopy. *Nature Methods* **2013**, *10* (6), 557–562. https://doi.org/10.1038/nmeth.2448.
